# The Effect of Carnosine on UVA-Induced Changes in Intracellular Signaling of Human Skin Fibroblast Spheroids

**DOI:** 10.3390/antiox12020300

**Published:** 2023-01-28

**Authors:** Gilda Aiello, Francesca Rescigno, Marisa Meloni, Beatrice Zoanni, Giancarlo Aldini, Marina Carini, Alfonsina D’Amato

**Affiliations:** 1 Department of Human Science and Quality of Life Promotion, Telematic University San Raffaele, 00166 Rome, Italy; 2 Department of Pharmaceutical Sciences, University of Milan, Via L. Mangiagalli 25, 20133 Milan, Italy; 3 VitroScreen, In Vitro Innovation Center, 20149 Milan, Italy

**Keywords:** 3D dermis spheroids, proteomics, carnosine, label free quantification, UVA

## Abstract

Dermis fibroblasts are very sensitive to penetrating UVA radiation and induce photo-damage. To protect skin cells against this environmental damage, there is an urgent need for effective compounds, specifically targeting UVA-induced mitochondrial injury. This study aimed to analyze the effect of carnosine on the proteome of UVA-irradiated human skin fibroblast, cultured in a three-dimensional (3D) biological system recapitulating dermal compartment as a test system to investigate the altered cellular pathways after 48 h and 7 days of culture with or without carnosine treatment. The obtained results indicate that UVA dysregulates Oxidative Phosphorylation, the Fibrosis Signaling Pathway, Glycolysis I and Nrf2-mediated Oxidative Stress Response. Carnosine exercises provide a protective function against the harmful effects of UVA radiation by activating the Nrf2 pathway with the upregulations of some ROS-detoxifying enzymes such as the glutathione S-transferase (GST) protein family. Additionally, carnosine regulates the activation of the Epithelial Adherens Junction and Wound Healing Signaling Pathway by mediating the activation of structural proteins such as vinculin and zyxin as well as fibronectin 1 and collagen type XVIII alpha 1 chain against UVA-induced changes.

## 1. Introduction

UV radiation has been described as one of the main physical factors that affects skin cells daily, resulting in inflammation, premature aging and cancer [1]. The UV light which naturally reaches the Earth’s surface contains UVB (280–320 nm) and UVA (320–400 nm) radiation. Each has unique properties, including energy, depth of penetration and biological effects. Among them, UVA is able to deeply penetrate into the skin dermis and it is considered the main cause of skin photoaging. UV-induced cellular damages are the main trigger of cellular senescence in skin, mainly in dermal fibroblasts, modulating extracellular matrix (ECM) deposition and remodeling.

Exposure of skin cells to UV radiation promotes the generation of reactive oxygen species (ROS) and subsequent lipid peroxidation, protein modifications and DNA damage, leading to the alteration of the antioxidant cellular system [2]. Contrary to the direct effect of ionizing radiations which corresponds to a direct ionization of DNA due to one electron ejection, UVA (and visible) light, by interaction with endogenous or exogenous photoreceptors, induces the formation of DNA damage through photomediated toxic reactions, characterized by low energy of the photons in respect to the ionizing radiations. As a result, proteins, lipids, and nucleic acids are altered by oxidative modifications which could induce the aggregation and formation of complex adducts. For example, reactive products of lipid peroxidation often bind to proteins causing a loss of antioxidant, proinflammatory and proapoptotic properties [3].

Skin mitochondria are highly involved in UVA skin damage. UVA induces the formation of mitochondrial ROS (mtROS) and mtDNA mutations and the perturbation of the electron transport chain, leading to ATP depletion and membrane depolarization [4]. Functional modules of mitochondria such as energy metabolism, biogenesis, fission and fusion are strongly affected by UV radiation. In this context, there is an urgent need for effective compounds, specifically targeting UVA-induced mitochondrial injury, to protect skin cells against this environmental damage. To date, several natural compounds endowed with antioxidants and carbonyl scavenger properties have been used to protect the skin against UV radiation [5,6]. Among them, carnosine, an endogenous β-alanyl-L-histidine dipeptide, has shown promising results in the prevention of Oxidative Stress modulation on the scaffold-free human dermis spheroids [7] and on UVA-irradiated nude mice skin [8]. Carnosine pretreatment in 3D-aged fibroblast resulted in modulation of important pathways such as free radical scavenging, apoptosis, mitochondrial function and ECM reorganization. In UVA-exposed nude mice, skin carnosine was able to neutralize the effects of reactive oxygen species by modifying proteins and impairing their functions. To further investigate the mechanism of action of carnosine in the prevention of UVA damage on human skin, the proteome of dermis spheroids of human fibroblast pretreated by UVA and carnosine was analyzed by quantitative mass spectrometry and network analyses. Three-dimensional cultured scaffold-free dermis spheroid represents the most suitable and micro-physiological model to mirror the complex homeostatic equilibrium and dynamical communicability between the cellular and extracellular space mimicking the physiological and pathological conditions of native tissue.

The application of VitroScreen ORA^®^ 3D scaffold-free dermis spheroids as a micro-physiological testing platform coupled with quantitative label-free proteomics enables a description of an in depth, in human cell-based physiological tissue system, the dynamic functional and structural response to photodamage induced by UVA exposure in the dermal stroma mirroring a native human dermis behavior. In particular, VitroScreen ORA^®^ spheroids are advanced tissue systems based on a bottom-up approach, able to better preserve the natural cell donor phenotype and consequently in the dermis deposition and architectural organization. The use of fibroblasts as target cells to study photodamage has been shown to be relevant by using targeted methods such as ELISA, qPCR or microscopy [9]. By exploiting the potential of a quantitative proteomics approach applied to the 3D spheroid model, the present work lays the foundations for its potential application to personalized care and medicine approaches as well as in dermatological research on aging and photoaging processes. Advanced analytical approaches based on genomics and proteomics allows a comprehensive study of the molecular pathways describing the alteration of protein phenotype by UV exposure and restored by carnosine treatment. By combining the advantages of the label-free quantitative proteomics with network analyses, the present study provides an in-depth investigation of the human dermis proteome signature after exposure to UVA radiation and the subsequent protective and antioxidant effect of carnosine.

## 2. Materials and Methods

### 2.1. Cell Source and Culture

Primary dermal fibroblasts isolated from a 59-year-old donor purchased from Innoprot (Derio, Spain), were cultured and amplified at early passages in CnT-PR F serum-free medium (CELLnTEC, Bern, Switzerland) in order to obtain 12 × 10^6^ cells.

### 2.2. VitroScreen ORA^®^ Dermis Spheroid Production

VitroScreen ORA^®^ dermis models were developed according to the VitroScreen internal procedure. In brief, primary human dermal fibroblasts were detached once 90% of confluence was reached. Cells were washed twice with DPBS 1X (Merck Life Science S.r.l., Milan, Italy) and incubated with T/E solution (Primary Detach Kit, Innoprot, Derio, Spain) for 5 min. Cells were counted, centrifuged and seeded in Akura^®^ Plate (InSphero AG, Schlieren, Switzerland) by ViaFlo Assist Plus (Integra Bioscience AG, Zizers, Switzerland) in order to obtain 10,000 cells/spheroids. Thanks to hanging drop technology, specific geometrical guidance allows cells to self-assemble forming round-shaped tridimensional scaffold-free spheroids. After 3 days in the hanging drop culture, spheroids were well formed and ready to be transferred into Akura^®^ V2 plate (InSphero AG, Schlieren, Switzerland).

### 2.3. Nucleic Acid Extractions for Nanostring Analysis and RNA QC

The 3D scaffold-free spheroids were lysed in proper buffer and the extraction of the nucleic acids was performed following the manufacturer’s instructions (RNeasy MicroKit by Qiagen, cat. 74004). RNA was eluted in RNAse-free water and stored at −80 °C until use. A total of 2 µL of RNA was used to perform QC by a 2100 Bioanalyzer (Agilent) on Agilent RNA 6000 Nano Chip with Agilent RNA 6000 Lader and Reagent. The method is based on capillary gel electrophoresis. All RNA samples were verified against the RNA acceptance criteria: a total RNA concentration ≥20 ng/µL, an RNA integrity number value ≥8.

### 2.4. Gene Expression Analysis Using Nanostring nCounter System

The hybridized sample was prepared on chip using nCounter MAX Prep Station and GPS transcripts were quantified using a NanoString Digital Analyzer (NanoString Technologies, Seattle, WA, USA). A total of 100 ng of RNA was used as sample input in a hybridization assay (NanoString Technologies, Seattle, WA, USA). Each hybridized sample was prepared on a cartridge using nCounter MAX Prep Station and an individual transcript of a biomarker signature was quantified using nCounter MAX Digital Analyzer (NanoString Technologies, Seattle, WA, USA). The control criteria for the NanoString analysis were imaging quality: >0.75; linearity: >0.95; limit of detection (LOD): (POS_E/LOD) >1 and binding density of 0.05 < BD < 2.25.

### 2.5. L-Carnosine Treatment and UVA Exposure

Following 72 h from transfer, the treated spheroid series were pre-incubated with 150 µM L-carnosine, previously observed as an efficacy dose to protect the dermis induced by aging [7], and added to the culture medium for 4 h. After pre-treatment, UVA series were prepared for UV exposure: the culture media with/or without treatment were discarded and DPBS1X was added to all tissues in order to avoid interference with UV exposure. ORA^®^ dermis spheroids were irradiated with UVA at a working distance of 12 cm, at 30 J/cm^2^ (Xenon Lamp Oriel Solar Simulator, Filter WG 335 3 mm) aiming to induce a perturbation of protein assessment in scaffold-free dermis spheroids; the dose was defined considering the complex physiology of the model and according to internal validated data [10]. Untreated and unexposed samples (NC) were rinsed with DPBS 1X and placed at RT in the dark in the same conditions of the exposed series. After UVA exposure, fresh medium with or without treatment was added to all series for 48 h and 7 days (D7) of recovery. All treated series were compared to NC and to untreated exposed samples (UVA). Fresh treatment was added every other day until 48 h and 7 days of culture. At each time point, three biological replicates of pooled 20 spheroids were collected, washed twice with cold DPBS 1X and allowed to settle to the bottom of conical tubes. After sedimentation, DPBS was discarded, and tissues were stored as pellets at −80 °C until the analysis.

### 2.6. Protein Extraction and In-Solution Trypsin Digestion

The cell pellets were resuspended in the solubilization buffer (8 M urea in 50 mM Tris–HCl, 30 mM NaCl, pH 8.5 and 1% protease inhibitor) and incubated on ice for 5–10 min. Tissue lysates were further homogenized by sonication in an ice bath, three times each for 15 s with 1 min intervals, using an ultra sonicator. Samples were centrifuged at 14,000× *g* for 20 min at 4 °C. The protein supernatant was collected into the new Eppendorf tube and the pelleted cell debris were discarded. Samples were stored at −80 °C until we used it for further experiments. The protein estimation was carried out by using the BCA assay. A total of 10 µg of proteins was diluted in 50 mM NH_4_HCO_3_ and then reduced with 5 mM DL-dithiothreitol (DTT, Sigma-Aldrich) for 30 min at 52 °C, shaken at 500 rpm and alkylated with 15 mM iodoacetamide (Sigma-Aldrich) for 20 min in the dark at room temperature. The trypsin digestion was performed in a 1:20 enzyme/protein ratio (*w*/*w*) (Trypsin Sequencing Grade; Roche, Monza, Italy) overnight at 37 °C. The obtained peptides were desalted using zip-tip C18, then dried and stored at −20 °C before the analysis.

### 2.7. High-Resolution LC-MS/MS Analysis and Data Elaboration

Tryptic peptides were analyzed using a Dionex Ultimate 3000 nano-LC system (Sunnyvale CA, USA) connected to an Orbitrap Fusion Tribrid Mass Spectrometer (Termo Scientifc, Bremen, Germany) equipped with a nano-electrospray ion source. Peptide mixtures were pre-concentrated onto an Acclaim PepMap 100–100 µm × 2 cm C18 and separated on an EASY-Spray column, 15 cm × 75 µm ID packed with Thermo Scientific Acclaim PepMap RSLC C18, 3 µm, 100 Å. The chromatographic separation was performed at 35 °C and the flow rate was 300 nL/min. Mobile phases were the following: 0.1% formic acid (FA) in water (solvent A); 0.1% FA in water/acetonitrile (solvent B) with 2/8 ratio. Peptides were pre-concentrated at 96% of solvent A and eluted from the column with the following gradient: 4% to 28% of B for 90 min and then 28% to 40% of B in 10 min, and to 95% within the following 6 min to rinse the column. The column was re-equilibrated for 20 min. Total run time was 130 min. One blank was run between triplicates to prevent sample carryover. MS spectra were collected over on an m/z range of 375–1500 at 120,000 resolutions, operating in the data-dependent mode, cycle time of 3 s. Higher-energy collisional dissociation (HCD) was performed with collision energy set at 35%. Each sample was analyzed in three technical triplicates. The resulting MS raw data from all the technical and biological replicates were analyzed by using MaxQuant software (version 1.6.2.3). The Andromeda search engine was used to identify MS/MS-based peptides and proteins in MaxQuant comprising a target-decoy approach with less than a 1% False Discovery Rate (FDR). In the present study we used the Uniprot_Homosapiens database. Trypsin was selected as the cutting enzyme, two missed cleavages and maximum number of five modifications per peptide were allowed. Methionine oxidation and acetylation (N terminus) was used as a variable modification. Carbamidomethylation was used as a fixed modification. The proteins were selected with a minimum of two peptides. For the label-free quantification of proteins, we applied the MaxLFQ algorithm. The match between the runs option was enabled and the remaining default parameters were permitted. The data are available on request from the authors. An open-source Perseus software (version 1.6.1.3; Max Planck Institute of Biochemistry, Germany) was used for the identification of statistically significantly, differentially regulated proteins. The interpretation and visualization of the results obtained from the MaxQuant software were performed by a two-sample *t*-test using Perseus (v1.6.1.3, Max Planck Institute of Biochemistry, Germany). Statistical parameters (*p* < 0.05; q < 0.05, q = FDR adjusted *p*-value) were set to identify the differentially expressed proteins between samples. Variabilities of biological replicates were measured with Pearson correlation coefficient values of the LFQ intensities. The differentially regulated proteins contained a minimum of two peptides and FDR adjusted *p*-value.

### 2.8. Protein Network Analysis

The protein network analysis related to significantly modulated proteins was carried out by Ingenuity Pathways Analysis (IPA) (QIAGEN Inc., June 2022, https://www.qiagenbioinformatics.com/products/ingenuitypathway-analysis). The statistical enrichment of involved pathways was performed by the right-tailed Fisher’s exact test, in correlation with QIAGEN Knowledge Base, assigning a *p*-value. The core analyses performed by IPA, using the differentially expressed proteins in the uploaded dataset, assess signaling pathways, molecular interaction networks and biological functions that can be likely perturbed. The overall activation/inhibition states of canonical pathways are predicted through a z-score algorithm. This z-score is used to statistically compare the uploaded dataset with the pathway patterns. The pathways are colored to indicate their activation z-scores: orange predicts a gain of function, while blue predicts a loss of function. The pathway is activated when molecules’ causal relationships with each other (i.e., activation edge and the inhibition edge between the molecules based on literature findings) generate an activity pattern for the molecules and the end-point functions in the pathway [11].

## 3. Results and Discussion

Dermis fibroblasts are one of the most representative cellular types of the human skin, are able to secrete structural and functional proteins, and are key molecules for the assembly of the deep stromal compartment. Due to their localization, dermis stromal fibroblasts are more protected from external factors than epidermal cells, forming the upper layer of the skin. As a result, dermis fibroblasts are very sensitive to penetrating UVA radiation. In this study, we evaluated the effect on a photo-damaged VitroScreen ORA^®^ dermis model by an omics approach for proteome remodeling after 48 h and 7 days of culture with or without carnosine treatment.

### 3.1. VitroScreen ORA^®^ Dermis Model to Study the UVA Dermis Damage and Protection Effect of Carnosine

VitroScreen ORA^®^ dermis models are miniaturized human biomimetic systems developed to mirror the physiology and the dynamic features of the native human dermis. The tissue engineered bottom-up approach allowed ORA^®^ models to mimic the natural dermis architecture in a 3D microscale: In this defined configuration, primary dermis fibroblasts at early passages aggregate according to their natural phenotype. During spheroids’ development, cells start to secrete endogenous key structural and functional molecules building a compact stromal ECM, without the addition of an exogenous support.

**Figure 1:**
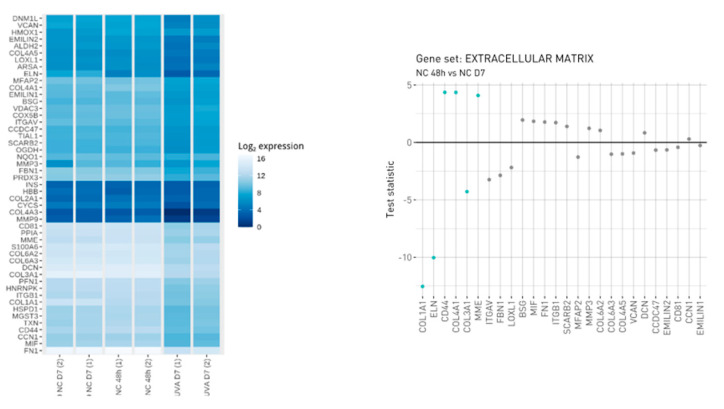
Visualization of observed expression levels. The left figure is a heatmap that displays the log2-transformed expression values and the right figure displays gene level statistics for genes within the manually curated gene sets. Statistics correspond to those from the differential expression analysis comparing NC_48 h vs. NC_D7 (analysis performed in triplicate, each replicate is a pool of 20 spheroids on 150–200 µm in size diameter).

In order to characterize the cellular model during the time culture, gene expression analysis was employed. The selected NanoString CodeSet included 59 genes, 49 of which were endogenous and related to ECM remodeling and to Oxidative Stress induced by photo-exposure. The remaining 10 genes were termed housekeeping genes and can potentially be used for purposes such as normalization and quality assessment. In addition to the probes targeting transcripts in the RNA samples, positive and negative control probes were also included in the CodeSet for assessing nonspecific background binding and to assess the effectiveness of hybridization. The hybridization reactions were successful as verified by NanoString quality control. The within-sample stability of the housekeeping genes was also assessed and a smaller subset of housekeeping genes was selected for the calculation of normalization scaling factors. These genes included NUBP1, EDC3, HDAC3 and PRPF38A. The observed expression of the CodeSet genes is shown in Figure 1. The ECM genes resulting in being differentially regulated during the defined experimental window such as HMOX1, COL4A1, CD44 and MME were upregulated while COL1A1, ELN and COL3A1 were downregulated, attesting to the induction of UVA-induced physiological aging of the extracellular matrix (Figure 1). The activation of metalloproteases such as MME suggests a disruption of ECM accompanied by a decrease in collagen isoform COL1A1 and COL3A1 during the time (48 h versus 7 days). However, the modulation of BSG and DCN, the latter binding collagen in the same region as the metalloprotease’s cleavage site, affords collagen protection against protease degradation indicating an ECM remodeling.

### 3.2. Protein Profiling of Human Dermis Spheroids Model after Exposure to UVA and Carnosine Treatment

**Figure 2:**
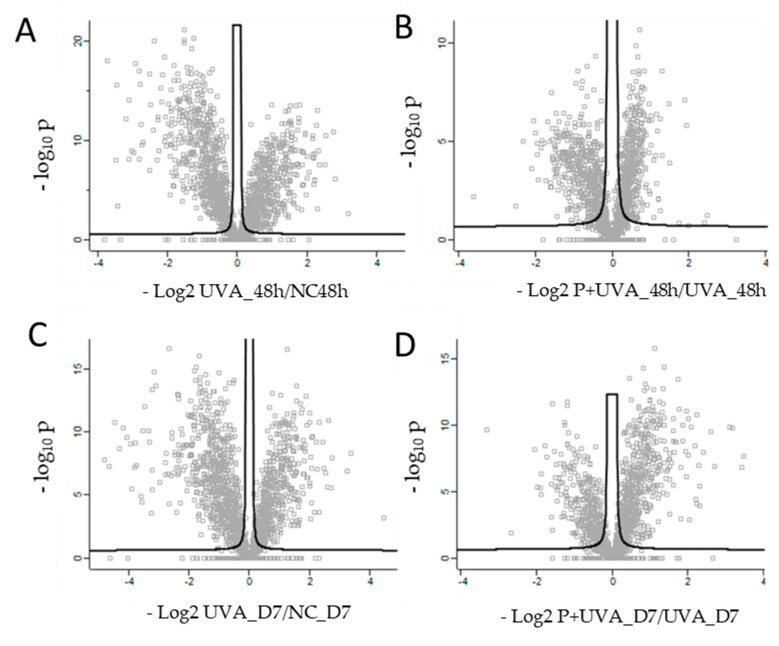
Scatter plots of log2 ratio on x-axis against -log10 *p*-value on y-axis of significantly quantified proteins resulted from two sided *t*-test. (**A**) Quantified proteins in human dermis spheroids after 48 h of UVA exposure (UVA_48hvsNC_48 h). (**B**) Quantified proteins in carnosine pre-treated dermis versus control at 48 h (P+UVA_48hvsUVA_48 h). (**C**) Quantified proteins in human dermis spheroids after D7 of UVA exposure versus control (UVA_D7vsNC_D7). (**D**) Quantified proteins in carnosine pre-treated dermis versus control at D7 (P+UVA_D7 vs. UVA_D7). Statistical parameters (*p* < 0.05; q < 0.05, q = FDR adjusted *p*-value) were set to identify the differentially expressed proteins between samples. Biological and technical triplicates were analyzed.

Thanks to a high biological relevance and their physiological profile, ORA^®^ dermis models are suitable as a tissue platform for in-depth investigation on UVA photo-damage induction and for evaluation of positive antioxidant role of carnosine in preserving tissue structural and functional integrity [7]. Spheroids showed a proteome capable of being induced by UVA external stimuli, described by 2912 proteins of which several were specifically differentially regulated. The exposure to 30 J/cm^2^ UVA radiations severely affected the basal proteomic profile of dermis spheroids inducing a modulation of key molecules related to Oxidative Stress Signaling, adhesion molecules and apoptotic events, showing a dynamic evolution during the time. The UVA treatment at 48 h resulted in the damage of spheroids characterized by 420 upregulated and 536 downregulated proteins, while the same treatment after D7 induced a variation of proteome described by 353 over expressed and 612 down expressed proteins (Figure 2A,C and Supplementary Table S1).

The UVA damage of spheroids was prevented by 150 µM carnosine added every other day, inducing the upregulation of 165 proteins and the downregulation of 303 proteins (48 h) and the upregulation of 320 proteins and the downregulation of 202 proteins (D7) (Figure 2B,D and Supplementary Table S1). Carnosine added as pre-treatment and during the recovery after UVA exposure, allowed a chemical protection against UVA photo-damage by preserving tissue viability, metabolism and ECM integrity, according to the duration of treatments. Specifically, network analyses showed UVA damages and their carnosine prevention, by specific functional modules, such as “Epithelial Adherens Junction Signaling”, “Oxidative Phosphorylation”, “Wound Healing Signaling Pathway” and “Nrf2-mediated Oxidative Stress Response” (Table 1) which are herein described.

### 3.3. UVA Induces the Modulation of Epithelial Adherens Junction Signaling

**Figure 3:**
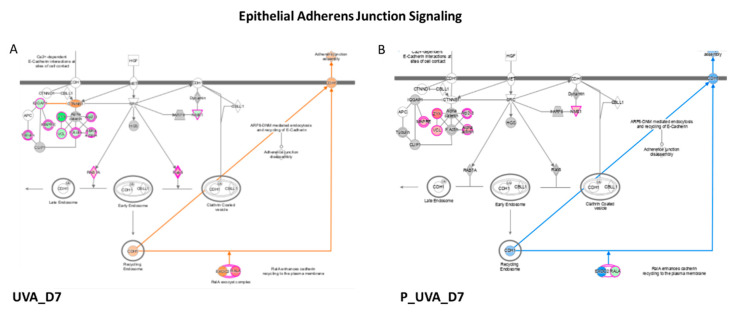
Epithelial Adherens Junction Signaling enhanced in UVA vs. control in (**A**) and decreased in (**B**) P_UVA vs. UVA (IPA). Red shows the increased features covered by our input database and green shows those decreased features. The orange color of the central hub indicates an upregulation of the module. For the node shapes: Knowledge: IPA Legend (salesforce-sites.com) (Qiagen, USA).

Network analyses, based on differentially regulated proteins, showed a negative modulation of epithelial Adherens Junction Signaling induced by UVA radiation after 48 h (z-score = −2.20) and 7 days (D7) (z-score = −2.35) of recovery (Table 1, Figure 3). The Adherens Junctions in epithelial cells are specialized structures belonging to the machinery of the cell–cell adhesion and consist of nectin and cadherin proteins, bound to actin cytoskeleton. In physiological and health conditions, actinin, zyxin, tubulins and myosins modulate cytoskeletal reorganization and actin polymerization, leading to the formation of Adherens Junctions. UVA radiation induces a downregulation of epithelial adhesion formation. Specifically, different actin isoforms such as actin-related proteins ARPC4 (log2 ratio = −0.93), ARPC2 (log2 ratio = −1.0) and ARPC1A (log2 ratio = −0.65), actin alpha 1, ACTN1 (log2 ratio = −0.88), actin alpha 4, ACTN4 (log2 ratio = −0.68), zyxin ZYX (log2 ratio = −2.13) and vinculin VCL (log2 ratio = −0.86), were downregulated by UVA at 48 h (Table 2). A similar trend was observed upon 7 days of recovery after UVA exposure, mostly for zyxin and vinculin whose log2 ratio dropped down to −2.42 and −1.11, respectively. The main function of zyxin is to form a bridge between the adhesion components of the cell membrane and the cytoskeleton, and its absence characterizes the early adhesions [12]. The UVA treatment resulted in a decrease of zyxin expression at 48 and 7 days after photo-damage (log2 = −2.13 and −2.42), while prevention of carnosine was efficient to restore its expression at both time points (ZYX, log2 ratio = 0.38 and 1.52), demonstrating the time-depending effects of carnosine on photo-damaged system. On the contrary, RAS-like proto-oncogene A, (RALA, log 2ratio = 1.53 at 48 h, log2 ratio =1.59 at D7) and the proteins belonging to the RAS oncogene family such as RAB7A (log 2ratio = 0.79 at 48 h, log 2ratio = 0,67 at D7) and RAB5C (log 2ratio = 0.72 at 48 h, log 2ratio = 0.82 at D7) were found overexpressed after both time of recovery after UVA exposure. RAS-mediated proliferative overdrive may induce replicative stress and activation of DNA damage responses [13]. This upregulation was observed to be modulated by carnosine reducing the overexpression of RALA (log2 ratio = −0.66 at at 48 h, log2 ratio = −0.78 at D7) and RAB7 (log2 ratio = −0.61 at 48 h, log2 ratio = −0.44 at D7). The impairment of cell adhesion and cellular motility was also confirmed by the activation of calpain protease at a long time point (D7) (z score = 0.91). Calpains are intracellular Ca^2+^-dependent cysteine proteases which regulate cell adhesion by modulating the spreading and motility of many cell types. It has been observed that calpain-deficient fibroblasts have enhanced membrane protrusion and filopodia formation [14]. In the meantime, the Integrin Signaling which shows a pivotal role in cell adhesion and adhesive signaling mostly for tissue repairing, was compromised by UVA exposure (z score = −1.71 at D7). Since the best protective effects of carnosine were observed after long-term treatment (7 days); from this point on, the discussion focuses on the description of the effects of carnosine treatment at D7.

### 3.4. UVA Induces the Modulation of Wound Healing Signaling Pathway

**Figure 4:**
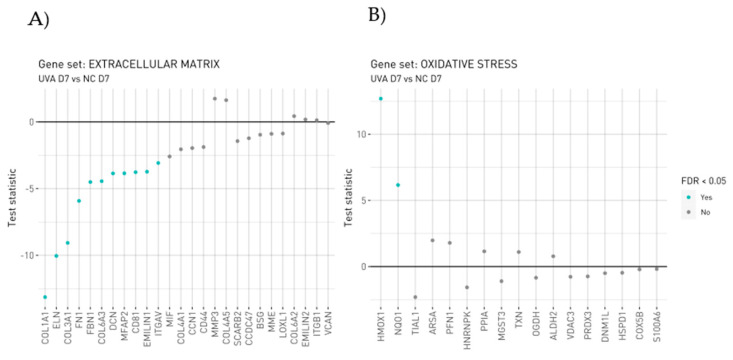
Gene level statistics for genes within the manually curated gene sets. (**A**) ECM genes altered by UVA_D7 vs. NC_D7. (**B**) Oxidative Stress genes altered by UVA_D7 vs. NC_D7. Statistics correspond to those from the differential expression analysis comparing UVA_D7 vs. NC_D7. Analysis was performed in triplicate; each replicate is a pool of 20 spheroids on 150–200 µm in size diameter.

Chronic UV exposure causes photoaging with profound alterations to the dermal connective tissue [15]. The morphological changes in skin fibroblasts exposed to UVA have already been demonstrated by scanning electron microscopy and immunofluorescence approaches. As fibroblasts are the main source of ECM, the functional changes in fibroblasts in photoaged skin are related to a reduction in collagen due to the inhibition of the polymerization of actin filaments accelerating the reduction in collagen synthesis [15]. In our study, both the UVA exposure reveals the dysregulation of collagen metabolism with the upregulation of proteins involved in the collagen degradation process, verified by gene expression too (Figure 4A). The upregulation of MMP-3 to the detriment of structural proteins as ELN, FN1, DCN, EMLIN1 and different isoform of collagen confirms the establishment of the well-known UVA-induced photoaging process negatively affecting ECM proteins. Wounds have chronic inflammation, impaired re-epithelialization to close the wound and abnormal dermal–epidermal connectivity that results in poor dermal–epidermal interaction, damaged microvasculature and abnormal collagen matrix deposition in the wound tissue [16]. Specifically, different isoforms of collagen, such as collagen type I, (COL1A1 log2 ratio = 0.96; COL1A2, log2 ratio = 0.49) III (COL3A1 log2 ratio = 0.45), IV (COL4A2, log2 ratio =1.00) V (COL5A1, log2 ratio = 0.31) and VI (COL6A1, log2 ratio = 1.71; COL6A2 log2 ratio = 2.02, COL6A3 log2 ratio = 4.48) resulted in being overexpressed in the carnosine treatment at D7 (Table 3). Among those, COL6A3 has the capability to direct matrix assembly and influence dermal cell behavior by interacting with other ECM molecules. Within ECM connective tissue, type VI collagen forms a highly branched filamentous meshwork that encircles the fibers of principal fibrillar collagens type I, II, and III [17].

### 3.5. UVA Induces Nrf2-Mediated Oxidative Stress Response

Considered as a “master regulator of the antioxidant response”, Nrf2 controls the transcription of genes encoding ROS-detoxifying enzymes and various other antioxidant proteins [18,19]. Nrf2 has a crucial role in the maintenance of cellular redox homeostasis by regulating the biosynthesis, utilization and regeneration of glutathione, thioredoxin and NADPH and by controlling the production of reactive oxygen species by mitochondria and NADPH oxidase. Under homeostatic conditions, Nrf2 affects the mitochondrial membrane potential, fatty acid oxidation, availability of substrates (NADH and FADH2/succinate) for respiration and ATP synthesis [20]. In fibroblasts, the activation of Nrf2 induces cellular senescence and its activation is qualified as a marker of the cancer-associated fibroblast phenotype. Previously published data showed that the Nrf2 activation in fibroblasts alters ECM protein expression and secretion by decreasing collagens type I and type III the most and several major proteoglycans (e.g., asporin, biglycan and versican) [21]. In UVB-treated keratinocytes, ROS-mediated apoptosis was reduced by the activation of Nrf2 [22]. In UVB-treated fibroblasts, instead, the defensive response of cellular death was induced by phosphorylated Akt by MAPK through the EGF receptor, independently to Oxidative Stress [23]. Indeed, in our work, UVA-exposed 3D spheroids have shown cell death (Table 1) due to the increasing concentration of ROS and downregulation of the Nrf2 pathway. Despite several publications reporting that PI3K/Akt pathway mediates the Nrf2 activation, in our dataset, the dephosphorylated AKT protein was found to be not regulated (Supplementary Table S1), whereas MAPK1 and MAP2K2 were down expressed (Table 4).

**Figure 5:**
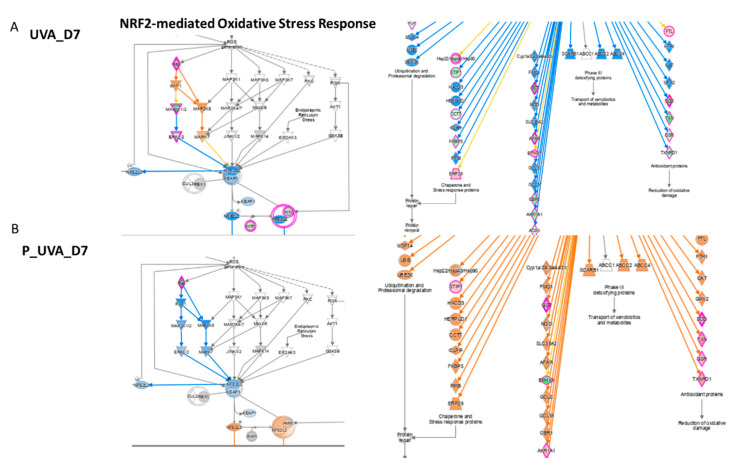
Genes involved in Nrf2 modulation after UVA exposure vs. control (**A**) after carnosine treatment (**B**) P_UVA vs. UVA (IPA). Red shows the increased genes, blue shows the decreased genes, grey shows the unaltered genes.

The UVA exposure at D7 modules the activation of Nrf2, responsible to the inducible expression of a group of detoxication enzymes, such as glutathione S-transferase including Glutathione S-transferase mu1 (GSTM1, log2 ratio = −2.43), Glutathione S-transferase mu3 (GSTM3, log2 ratio = −1.84), Glutathione S-transferase omega1 (GSTO1, log2 ratio = −1.67), Glutathione S-transferase pi1 (GSTP1, log2 ratio = −1.77) and Glutathione disulphide reductase (GSR, log2 ratio = −0.72) (Figure 5 and Table 4). Our finding underlines the role of Nrf2 as a truly pleiotropic transcription factor by playing an important role in the downregulation of several glutathione S-transferases, which are the enzymes mediating the elimination of reactive oxygen species (ROS). Reduced glutathione (GSH) interacts directly with ROS to form oxidized glutathione (GSSG).

GSH is also a co-substrate for several detoxication enzymes such as glutathione peroxidase (GPx) and glutathione S-transferase (GST). Regeneration of oxidized GSH (GSSG) is mediated by an enzyme, glutathione reductase (GSR), that in this way participates in maintaining redox homeostasis [24]. Decreased GSH concentration, due to UVA-stimulated ROS production, results in the alteration of redox homeostasis in cells.

Moreover, the evidence of strong UVA-induced Oxidative Stress is found in the downregulation of Superoxide dismutase1 (SOD1, log2 ratio = −2.19), thioredoxin (TXN, log2 ratio = −1.87) and thioredoxin reductase1 (TXNRD1 log2 ratio = −0.93), proteins involved in redox cellular homeostasis and verified by gene expression too Specifically, UVA induced the increment of HMOX1, considered as critical ferroptosis-related gene (Figure 4B).

However, this trend has been observed to be reversed by carnosine after 7 days of treatment which inhibits UV-induced damage by restoring the proteins’ level as the following: Glutathione S-transferase mu1 (GSTM1, log2 ratio = 1.00), Glutathione S-transferase mu3 (GSTM3, log2 ratio = 0.71), Glutathione S-transferase omega1 (GSTO1, log2 ratio = 1.24), Glutathione S-transferase pi1 (GSTP1, log2 ratio = 1.10) and Glutathione disulphide reductase (GSR, log2 ratio = 0.77). As well as a strong upregulation, the irradiated and treated series for 7 days was observed for Superoxide dismutase1 (SOD1, log2 ratio = 1.30), thioredoxin (TXN, log2 ratio = 1.19) and thioredoxin reductase1 (TXNRD1 log2 ratio = 1.09). Specifically, the variation of SOD1 and TXT is involved in the downregulation of the quantity of reactive oxygen species pathway (z score = −2.75) induced by carnosine. The activation of Nrf2 in fibroblasts also induces cellular senescence due to the activation of plasminogen activator inhibitor-1 being a key inducer of the senescence program which induces a deposit of a senescence-promoting matrix [21]. It has been also observed that Nrf2 mediated the fibroblast senescence with a faster re-epithelialization and accelerated wound closure. Additionally, UVA induces the activation of the Mitogen Activated Protein Kinase (MAPK) pathway which was restored by carnosine treatment. In addition, carnosine induced a reduction of apoptosis (Table 1) at both incubation time and the upregulation of the Nrf2 pathway to reduce Oxidative Stress and senescence too.

### 3.6. UVA Regulates the Glycolysis and the Oxidative Phosphorylation

In excess, oxidation can provoke metabolic failure, compromising cell viability by inactivating enzymes of Glycolysis and the Krebs cycle. ROS can inhibit Glycolysis allowing the cells to divert flux into the pentose phosphate pathway (ox-PPP) to promote NADPH synthesis and protect against Oxidative Stress [25].

The indirect evidence of the ROS effect evoked by UVA radiation after 7 days of carnosine treatment concerns the downregulation of multiple glycolytic enzymes, including glyceraldehyde 3-phosphate dehydrogenase (GAPDH, log2 ratio = −1.24), pyruvate kinase M2 (PKM, log2 ratio = −1.64), phosphofructokinase (PFKL, log2 ratio = −1.88) and pyruvate kinase M1/2 (PKM, log2 ratio = −1.64). It is well known that Glycolysis can divert to combat the Oxidative Stress. ROS inhibits multiple glycolytic enzymes, including glyceraldehyde 3-phosphate dehydrogenase, pyruvate kinase M2 and phosphofructokinase-1, directly to target cysteine residues [25]. While the nicotinamide adenine dinucleotide (NADH/NAD+) drives ATP production in the cytosol by Glycolysis and in the mitochondria by Oxidative Phosphorylation, nicotinamide adenine dinucleotide phosphate (NADPH/ NADP+) is mainly involved in the defense against reactive oxygen species [26,27]. Consistently, glycolytic inhibition could promote flux into the oxidative arm of the pentose phosphate pathway to generate NADPH. NADPH provides the reducing power that fuels the protein-based antioxidant system and its concentration is regulated by the pentose phosphate pathway. In addition, NADPH is the donor of the reductive potential to thioredoxins and glutathione, which in turn neutralize ROS. NADPH is, in fact, consumed by glutathione reductase (GSR) to recycle oxidized glutathione (GSSG). A metabolic switch from Glycolysis to Oxidative Phosphorylation or Oxidative Stress possibly correlated to ROS generation has been already observed [28]. NAD(P)H biomarker showed an early change at 30 min after exposure in dermal fibroblasts, while a significant change in epidermal keratinocytes was only observed at 2 h after exposure to UVA [28]. The results here highlighted the activation, mediated by UVA, of Oxidative Phosphorylation (OXPHOS) with the upregulation of several core subunits of NADH: ubiquinone oxidoreductase, complex I. Considering its heterogeneity, due to its 13 subunits, complex I is very susceptible to oxidative damage. Here, 10 subunits were detected as upregulated upon 7 days of treatment after UVA radiation. Specifically, NDUFS2 (core subunit 2 log2 ratio = 0.253), NDUFS3 (core subunit 3 log2 ratio = 0.658), NDUFS8 (core subunit 8 log2ratio = 0.93), NDUFV2 (core subunit V2 log2 ratio = 0.607), NDUFA13 (subunit A13 log2 ratio = 2.653), NDUFA2 (subunit A2 log2 ratio = 0.663), NDUFA5 (subunit A5 log2 ratio = 1.018), NDUFA8 (subunit A8 log2 ratio = 1.562), NDUFA9 (subunit A9 log2 ratio = 0.429) and NDUFB10 (subunit B10, log2 ratio = 1.413) (Table 5). Many therapeutic properties of carnosine as a pharmacological and cosmetic substance have been described either on 3D fibroblast cells or animals [29]. By penetrating at multilayer/spheroid fibroblast, carnosine is able to exert it effect on the mitochondrial metabolism, inducing an enhancement of glycolytic enzymes characterized by an increase in log 2 ratio as follows: (GAPDH, log2 ratio = 0.63), glucose-6-phosphate isomerase (GPI, log2 ratio = 1.28) and phosphoglycerate kinase1 (PGK1, log2 ratio = 0.78).

### 3.7. UVA Radiations Regulates the Sirtuin Signaling Pathway

The network analysis reveals the modulation of 44 genes involved in the Sirtuin pathway which overall is downregulated (z score = −1.25) after 7 days related to the control. Sirtuins (SIRTs) comprise one of four classes of histone deacetylases (HDACs; I–IV) that play important roles in a variety of cellular functions. Here, three isoforms of linker histone, namely H1.0 (log 2 ratio = 1.91), H1.4 (log2 ratio = 1.50), H1.5 (log2 ratio = 1.94), were found to be strongly upregulated upon the UVA exposure. Several studies have demonstrated that SIRT1 levels decrease with age in dermal fibroblasts isolated from female donors who ranged from 20–67 years old [30]. Specifically, the case for SIRT1 involvement in UVB-mediated DNA damage has been observed in several in vitro experiments using human fibroblasts. The UVB radiation has been shown to decrease SIRT1 protein levels [31]. However, although SIRT1 has been observed to decrease in in vitro studies, Lang et al. showed that SIRT4 levels increase in fibroblasts exposed to UVB radiation and this correlates with an increase in cellular senescence [32]. On the basis of these observations, the role of SIRT1 as a tumor promoter or suppressor in UVB-induced cancer initiation is unclear and might vary with cell/tissue type or protein levels. However, the finding of this study on 3D spheroids agrees with those observed in nude mice skin upon UVA exposure [8].

## 4. Conclusions

UVA exposure is a powerful attack on the skin, in particular, the dermal compartment determining a non-reversible dermal damage affecting the skin elasticity and inducing photoaging and photo-carcinogenesis. Within the very early events of the photoaging process, the ROS generation, by promoting mitochondrial electron transport chain damage, can be considered the most relevant. The present study, based on a combination of network analyses and high-resolution mass spectrometry applied to advanced micro-physiological scaffold-free 3D human spheroids underlines the protective role of carnosine against UVA-induced damages and reveals wide mechanisms of action on several pathways and molecular signaling. Indeed, the response to carnosine treatment highlighted the modulation of several pathways, in particular, related to mitochondrial protection. The modulation of multiple pathways such as Oxidative Phosphorylation, Glycolysis I and the Nrf2-mediated Oxidative Stress Response, highlight both the role of mitochondrial dysfunctions induced by UVA and the protective role of carnosine against them; as well, it was associated with a positive effect on fibrillin, which as early marker of dermal matrix remodeling, confirms the response to UVA stimulus and the protective role of carnosine.

In conclusion, due to the advanced micro-physiological dermal spheroids system conjugated by genomics and proteomics, it has been possible in a relatively short experimental window to mirror the cascade of complex events that follow UVA exposure and to observe the recovery from the damage as it physiologically occurs in vivo thanks to an antioxidant reference molecule, carnosine, that was confirmed and able to offer the protection of dermis against early and delayed UVA damages at the dermal level.

## Figures and Tables

**Table 1:** Functional modules evoked by UVA exposure. Positive z-score indicates the activation pathway; negative z-score indicates the downregulation of the pathway (the analysis was performed by IPA (Qiagen) using the quantitative LFQ dataset).

	Z-Score			
Canonical Pathways	UVA_48h	P_UVA_48h	UVA_D7	P_UVA_D7
Oxidative Phosphorylation	5.39	−1.16	4.71	−1.67
Hepatic Fibrosis Signaling Pathway	2.41	−1.60	1.57	−2.31
Wound Healing Signaling Pathway	1.53	−2.53	1.61	−3.16
Estrogen Receptor Signaling	3.14	−2.11	1.89	−2.89
Macropinocytosis Signaling	2.45	−2.45	2.24	−2.45
GP6 Signaling Pathway	2.14	−1.63	0.91	−2.12
Pulmonary Fibrosis Idiopathic Signaling Pathway	1.23	−2.31	1.00	−2.53
G Beta Gamma Signaling	2.11	−2.65	0.91	−2.65
Gαi Signaling	1.90	−2.45	1.51	−2.45
Regulation of eIF4 and p70S6K Signaling	1.67	−2.45	1.73	−2.45
p70S6K Signaling	1.90	−2.45	1.39	−2.24
Thrombin Signaling	1.51	−2.83	0.91	−2.65
NF-κB Activation by Viruses	1.89	−2.24	1.41	−2.24
Regulation of Cellular Mechanics by Calpain Protease	2.12	−1.89	0.91	−2.45
Glioma invasiveness Signaling	1.89	−2.45	0.63	−2.24
HGF Signaling	1.16	−2.45	0.28	−2.45
UVA-induced MAPK signaling	1.13	−2.00	1.13	−2.00
CDK5 Signaling	1.51	−2.24	0.26	−2.24
PEDF Signaling	1.14	−1.63	0.71	−2.24
Fc Epsilon RI signaling	−1.13	−2.-24	0.38	−2.-24
Glycolysis I	−3.46	1.41	−3.61	2.83
PTEN Signaling	−1.73	2.65	−1.16	2.45
Epithelial Adherens Junction Signaling	−2.20	1.27	−2.35	2.00
CLEAR Signaling Pathway	−1.67	1.70	−1.41	2.45
Gluconeogenesis I	−2.11	2.45	−2.11	2.83
Sirtuin Signaling Pathway	−0.85	−2.32	−1.26	−0.83
Apotosis	1.25	−0.94	2.19	−2.82

**Table 2:** Proteins involved in the Epithelial Adherens Junction Signaling. Log2 ratio ≥ 0.6 indicates upregulation, log2 ratio ≤ − 0.6 represents downregulation.

Epithelial Adherens Junction Signaling			UVA_48 h	P_UVA_48 h	UVA_D7	P_UVA_D7
Gene Name	Protein Name	Accession Number	Log Ratio	Log Ratio	Log Ratio	Log Ratio
RALA	RAS-like proto-oncogene A	P11233	1.53	−0.66	1.59	−0.78
RAB7A	RAB7A, member RAS oncogene family	A0A158RFU6	0.79	−0.61	0.67	−0.44
RAB5C	RAB5C, member RAS oncogene family	A0A024R1U4	0.72	−0.38	0.82	−0.30
ARPC4	Actin-related protein 2/3 complex subunit 4	P59998	−0.93	0.46	−0.53	0.26
ARPC1A	Actin-related protein 2/3 complex subunit 1A	Q75MY0	−0.65	0.25	−0.76	0.31
ARPC2	Actin-related protein 2/3 complex subunit 2	Q53R19	−1.00	0.36	−0.40	0.36
NME1	NME/NM23 nucleoside diphosphate kinase 1	P15531	−1.01	0.62	−0.77	0.64
MAPRE1	Microtubule-associated protein RP/EB family member 1	Q15691	−1.29	0.62	−1.18	0.70
ACTN4	Actinin alpha 4	A0A0S2Z3G9	−0.68	0.54	−0.69	0.79
ACTN1	Actinin alpha 1	A0A024R694	−0.88	0.57	−0.83	0.87
VCL	Vinculin	A0A024QZN4	−0.86	0.35	−1.11	0.87
ZYX	Zyxin	Q15942	−2.13	0.38	−2.42	1.52

**Table 3:** Proteins involved in the Wound Healing Signaling Pathway.

Wound Healing Signaling Pathway			UVA_48 h	P_UVA_48 h	UVA_D7	P_UVA_D7
Gene Name	Protein Name	Accession Number	Log Ratio	Log Ratio	Log Ratio	Log Ratio
COL1A1	Collagen type I alpha 1 chain	D3DTX7	1.99	−0.99	0.96	−1.27
COL3A1	Collagen type III alpha 1 chain	P02461	0.79	0.53	0.45	0.00
COL1A2	Collagen type I alpha 2 chain	A0A087WTA8	1.20	0.00	0.49	0.00
COL5A1	Collagen type V alpha 1 chain	A0A024R8E5	0.99	0.24	0.31	0.00
COL15A1	Collagen type XV alpha 1 chain	P39059	−1.74	0.51	−2.35	0.48
LAMA2	Laminin subunit alpha 2	A0A087WYF1	0.60	0.00	0.00	0.00
COL6A2	Collagen type VI alpha 2 chain	P12110	2.30	−1.04	2.02	−1.26
COL6A1	Collagen type VI alpha 1 chain	A0A087X0S5	1.77	−0.50	1.71	−0.93
RRAS	RAS related	A0A024QZF2	0.93	−0.41	1.05	−0.85
RALA	RAS-like proto-oncogene A	P11233	1.53	−0.66	1.59	−0.78
RAP1B	Rap1b Member of RAS oncogene family	P61224	1.28	−0.84	1.04	−0.75
ITGB1	Integrin subunit beta 1	P05556	1.36	−0.74	1.30	−0.71
RAC1	Rac family small gtpase 1	A4D2P1	0.81	−0.88	0.52	−0.70
COL4A2	Collagen type IV alpha 2 chain	P08572	0.75	0.00	1.00	−0.28
MAP2K2	Mitogen-activated protein kinase kinase 2	P36507	−1.49	0.00	−2.00	0.00
MAPK1	Mitogen-activated protein kinase 1	Q1HBJ4	−0.73	0.00	−0.94	0.00
STAT1	Signal transducer and activator of transcription 1	P42224	−0.95	0.00	−0.60	0.00
RRAS2	RAS related 2	P62070	0.77	−1.04	0.95	0.00
FN1	Fibronectin 1	A0A024R462	−1.08	0.00	−1.28	0.28
COL18A1	Collagen type XVIII alpha 1 chain	D3DSM4	−1.36	0.58	−1.38	0.55
COL6A3	Collagen type VI alpha 3 chain	B7ZW00	3.19	−3.60	4.48	−2.66

**Table 4:** Proteins involved in the Nrf2-mediated Oxidative Stress Response.

Nrf2-Mediated Oxidative Stress Response			UVA_48h	P_UVA_48h	UVA_D7	P_UVA_D7
Gene Name	Protein Name	Accession Number	Log Ratio	Log Ratio	Log Ratio	Log Ratio
AKR1A1	Aldo-keto reductase family 1 member A1	V9HWI0	−0.94	0.40	−1.32	0.83
CLPP	Caseinolytic mitochondrial matrix peptidase proteolytic subunit	Q16740	0.73	−0.33	0.58	0.00
EPHX1	Epoxide hydrolase 1	R4SBI6	1.30	−1.18	0.97	−0.89
GSTM1	Glutathione S-transferase mu 1	X5DR03	−1.29	−0.61	−2.43	1.00
GSTM3	Glutathione S-transferase mu 3	Q6FGJ9	−1.20	0.00	−1.84	0.71
GSTO1	Glutathione S-transferase omega 1	V9HWG9	−1.69	0.73	−1.67	1.24
GSTP1	Glutathione S-transferase pi 1	V9HWE9	−1.44	0.51	−1.77	1.10
GSR	Glutathione-disulphide reductase	V9HW90	−0.92	0.64	−0.72	0.77
RAP1B	RAP1B, member of RAS oncogene family	P61224	1.28	−0.84	1.04	−0.75
RALA	RAS-like proto-oncogene A	P11233	1.53	−0.66	1.59	−0.78
RRAS	RAS related	A0A024QZF2	0.93	−0.41	1.05	−0.85
RRAS2	RAS related 2	P62070	0.77	−1.04	0.95	0.00
SOD1	Superoxide dismutase 1	V9HWC9	−1.91	0.62	−2.19	1.30
TXN	Thioredoxin	H9ZYJ2	−1.22	0.00	−1.87	1.19
TXNRD1	Thioredoxin reductase 1	Q16881	−1.15	0.74	−0.93	1.09
SOD2	Superoxide dismutase 2	P04179	1.44	0.00	1.65	0.00
MAPK1	Mitogen-activated protein kinase 1	Q1HBJ4	−0.728	0.00	−0.939	0.00
MAP2K2	Mitogen-activated protein kinase kinase 2	P36507	−1.488	0.00	−1.996	0.00

**Table 5:** Proteins involved in the Glycolysis pathway.

Glycolysis			UVA_48 h	P_UVA_48 h	UVA_D7	P_UVA_D7
Gene Name	Protein Name	Accession Number	Log Ratio	Log Ratio	Log Ratio	Log Ratio
ALDOA	Aldolase, fructose-bisphosphate A	P04075	−1.33	0.63	−1.61	1.02
ALDOC	Aldolase, fructose-bisphosphate C	A0A024QZ64	−1.30	0.76	−1.70	0.96
ENO1	Enolase 1	A0A024R4F1	−1.24	0.66	−1.59	0.92
ENO2	Enolase 2	Q6FHV6	−1.26	0.74	−2.14	1.58
GAPDH	Glyceraldehyde-3-phosphate dehydrogenase	V9HVZ4	−1.14	0.34	−1.24	0.63
GPI	Glucose-6-phosphate isomerase	K7EIL4	−1.66	0.71	−1.85	1.28
PFKL	Phosphofructokinase, liver type	P17858	−1.22	−1.07	−1.88	0.00
PFKM	Phosphofructokinase, muscle	P08237	−1.00	0.00	−0.93	0.00
PGAM1	Phosphoglycerate mutase 1	Q6FHU2	−0.88	0.46	−1.14	0.52
PGK1	Phosphoglycerate kinase 1	V9HWF4	−1.51	0.54	−1.82	0.78
PKM	Pyruvate kinase M1/2	A0A024R5Z9	−1.00	−0.23	−1.64	0.00
TPI1	Triosephosphate isomerase 1	V9HWK1	−1.35	0.85	−1.61	1.35

## Data Availability

Data is contained within the article and supplementary material.

## Supplementary Materials

The following supporting information can be downloaded at: https://www.mdpi.com/article/10.3390/antiox12020300/s1, Table S1. List of quantified proteins obtained by MaxQuant software.
